# Proposing a Caputo-Land System for active tension. Capturing variable viscoelasticity

**DOI:** 10.1016/j.heliyon.2024.e26143

**Published:** 2024-02-13

**Authors:** Afnan Elhamshari, Khalil Elkhodary

**Affiliations:** a The Robotics, Control, and Smart Systems Program, The American University in Cairo, 11835, New Cairo, Egypt; b The Department of Mechanical Engineering, The American University in Cairo, 11835, New Cairo, Egypt

**Keywords:** Active tension, Variable cardiac viscoelasticity, Fractional-order systems, Quick stretch and release experiments, Caputo's fractional derivatives, Mean square error (MSE)

## Abstract

Accurate cell-level active tension modeling for cardiomyocytes is critical to understanding cardiac functionality on a subject-specific basis. However, cell-level models in the literature fail to account for viscoelasticity and inter-subject variations in active tension, which are relevant to disease diagnostics and drug screening, e.g., for cardiotoxicity. Thus, we propose a fractional order system to model cell-level active tension by extending Land's state-of-the-art model of cardiac contraction. Our approach features the (left) Caputo derivative of six state variables that identify the mechanistic origins of viscoelasticity in a myocardial cell in terms of the thin filament, thick filament, and length-dependent interactions. This proposed CLS is the first of its kind for active tension modeling in cells and demonstrates notable subject-specificity, with smaller mean square errors than the reference model relative to cell-level experiments across subjects, promising greater clinical relevance than its counterparts in the literature by highlighting the contribution of different cellular mechanisms to apparent viscoelastic cell behavior, and how it could vary with disease.

## 1 Introduction

Cardiac modeling is crucial to understanding cardiac functions in both health and disease. Specifically, it sheds light on pathological and physiological cardiac processes that are difficult to quantify in-vivo or in-vitro, connecting those processes to their underlying cellular mechanisms [[1], [2], [3], [4], [5], [6]], as well as serving to predict cardiotoxic drug interactions on a patient-specific basis [[7], [8], [9]].

In this paper, we focus on active tension modeling. It refers to the sarcomere length-tension relation that develops during a cardiac cycle, which is crucial to understanding cardiac function on the organ level [10]. In humans, active tension can be attributed to three main processes that unfold simultaneously within a cardiac cell: thin filament kinetics, thick filament kinetics, and the Frank-Starling law (length-dependency of tension) [11]. Thin filament kinetics refers to the interactions between the intracellular calcium ions and the proteins enclosed in the cell, including Troponin C, Troponin I, and tropomyosin, which regulate the myosin binding sites on actin [12,13]. Thick filament kinetics, on the other hand, relates to how cross-bridges form, attach, and detach from the thin filaments to generate a contractile force [14,15]. Conversely, the causes of the Frank-Sterling law are not yet fully understood. The dependency of the change in intracellular calcium levels on sarcomere length is theorized to be a leading cause behind this law [1,16]. As such, the law focuses on the cardiac ventricular systole, where length-dependent activation relates to intracellular calcium levels in the sarcomere.

Various computational models have been put forward to explain one or more interactions of active tension in human or animal cardiac cells. One of the first models advanced to capture saturated muscle cross-bridge interactions is Huxley's model. It is, to date, at the basis of most muscle models. The model comprises a simple population-dynamics-based Partial Differential Equation (PDE) system, which assumes linear elastic mechanics [17]. It however fails to properly capture the role of thin and thick filament kinetics on cross-bridge dynamics. More complex PDEs have thus been advanced by researchers to better capture elastic cross-bridge dynamics on a cellular level [18]. Similarly, stochastic modeling has attracted much research to elucidate thin filament interactions in mice, rats, and humans. For instance, the authors of [19] proposed an approach based on Ordinary Differential Equation (ODE) systems with a probabilistic Boltzmann model. Although their model accurately captures the binding interaction between tropomyosin and cross bridges, it features higher-order derivatives, making it difficult to implement and calibrate for its many variables [19]. Alternatively, the authors of [20] proposed an ODE stochastic model based on a Markovian chain, while the authors of [21] proposed using Monte Carlo simulations to model the changes in cross-bridge dynamics. Again, both models provide advantageous mechanistic understanding but require computationally expensive implementations. Moreover, these models can only lend insight into a single interaction, failing to represent all relevant aspects of active cardiac tension.

Conversely, Land's model of cardiac contractility is a recent model that holistically explains active cardiac tension using a system of ODEs that integrates thin, thick, and length-dependent interactions based on human cardiac single-cells [22]. It has been used as a mathematical basis for active contraction of the human cardiac myocyte [23] and will thus serve as a basis for our research in this paper.

We note that Land's model, like all the above cardiac models, was developed assuming that cardiac cells and their internal states change with respect to time according to an integer-order system, ODEs, or PDEs, which is not necessarily true for viscoelastic behaving materials. Indeed, cardiac cells are viscoelastic in nature [24], and the cytoskeleton has been shown to be a great contributor to cellular viscoelasticity [25,26]. If a disease adversely affects their viscoelasticity, ventricular diastolic and systolic dysfunction can often arise, rendering this a clinically relevant phenomenon [27,28]. In fact, much research has been conducted that characterizes this viscoelastic behavior, using different versions of Hill's equations [29], systems of quasilinear functions [30], or, more powerfully, by implementing systems of Fractional-order Differential Equations (FDE) [[31], [32], [33]]. This latter direction is the one we will pursue in this paper to advance Land's model and allow it to distinguish contributions from underlying kinetic variables to resultant cellular viscoelasticity and mechanics while respecting the known networks of interaction.

FDEs are based on fractional calculus, which is a branch of mathematics that deals with non-integer order derivatives and integrals. It has been proven to be a powerful modeling tool in many fields, such as control theory [34], medical electronics [35], cell-biology [[36], [37], [38]], topology [39], environmental science [40,41], and other viscoelastic applications [[42], [43], [44], [45], [46], [47], [48], [49]]. As noted above, fractional calculus is especially helpful in describing viscoelastic behavior for its non-local definition of derivatives, which feature finite time-history integrals [50]. In particular, the descriptive power of fractional derivatives in viscoelasticity is equivalent to that of an infinite tree of spring and dashpot elements [33], yet they do not necessitate the introduction of any new term into the governing differential system of equations, rendering them simpler to formulate and interpret.

In macroscale cardiac modeling, FDEs were successfully used by the authors of [51] to describe the viscoelastic behavior of the aortic valve as an alternative to the quasilinear functions. They showed that the aortic behavior could be better fitted using non-integer-order equations. As an interesting application, FDEs were also shown to be especially helpful in optimizing a subject-specific drug delivery system based on the subject's needs [52]. This subject-specificity of FDEs could render Land's model more capable than its current ability only to characterize average responses.

Therefore, in this study, we develop an FDE-based rewrite of Land's model that accurately portrays active tension in human single cells, capturing their viscous response successfully and facilitating future subject-specific modeling at the organ level. Specifically, the descriptive power of the FDEs introduced herein permits a simple characterization of observable cell-level mechanics (e.g., obtained from in-vitro tests), and inter-subject variabilities thereof, while easily distinguishing viscoelastic contributions from known kinetic mechanisms that underlie the apparent cell properties.

Our paper is divided as follows. Our methods section explains our proposed Caputo-Land System (CLS) and the computational steps that implement and calibrate it per Land's experimental data. Then, our Results and Discussion section details our preliminary findings compared to the original Land model and highlights the potential of the CLS as an in-silico guide for in-vitro disease diagnostics and drug screening. Finally, our conclusion summarizes how the proposed CLS better captures cardiac functionality by accounting for viscous effects within a mechanistic framework.

## 2 Methods

### 2.1 Fractional derivatives

Fractional calculus is a branch of mathematics that generalizes differentiation and integration to non-integer orders. Specifically, a fractional derivative of a function represents the rate of change of that function with respect to a fractional order, yielding a curve that lies arbitrarily between that of the function and that of its integer derivative. Fractional derivatives found application in various fields, as noted in the Introduction. The literature has many definitions of fractional derivatives, depending on the order of applying Euler's Gamma function (Γ), which is key to generalizing factorial non-integer functions and featuring non-integer derivatives [53]. Caputo's derivative is a commonly used definition because it is the simpler alternative to solve numerically, is more accurate, and allows for non-zero initial conditions, which we need in this implementation [54]. Other definitions include the Riemann-Liouville and Grunwald-Letnikov fractional derivatives [53], which we will not pursue herein.

Caputo's left definition (DaCtα) of a fractional derivative of a function *f*(*t*), where t∈[a,b], is given by equations (1), (2) below,

(1) DaCtαf(t)={1Γ(n−α)∫atf(n)(τ)(t−τ)∝+1−ndτ,n−1≤α<n,dndtnf(t)α=n.

(2) $$\Gamma \left(z\right)=\lim_{n\to \infty }\frac{n!n^{z}}{\prod_{k=0}^{n}\left(z+k\right)}\;Re\left(z\right)>0$$

In equation (1), *α* designates the fractional order of the derivative, and Γ the Euler gamma function. As the finite time-history integral in equation (1) indicates, fractional derivative operators are non-local. Non-locality implies that the value of the fractional derivative at time *t* depends on the value of the *n*-th derivative of *f*(*t*) integrated between *a* and *t*. This time-history integral is characteristic of modeling viscoelasticity [55]. For their non-locality, we posit that FDEs are best suited to capture the kinetic processes at the cell level, which govern the different rates of sarcomere stretch and release, and which span and interact over extended/dissimilar time scales.

### 2.2 Our proposed Caputo-Land System (CLS)

We here describe our proposal to rewrite the Land Model using fractional derivatives to create what we call the Caputo-Land System (CLS). Originally, Land's model captured active cardiac tension via a system of six differential equations which evolve six states. These six states are:

o **ζ**_**w**_: Cross-bridge distortion, pre-power-stroke.
o **ζ**_**s**_: Additional cross-bridge distortion, post-power-stroke.
o **W**: Weakly bound cross-bridges, pre-power-stroke.
o **S**: Strongly bound cross-bridges, post-power-stroke.
o **CaTRPN**: The fraction of Troponin C units with bound calcium.
o **B**: The fraction of blocked myosin binding sites on actin.

Four of these six states represent structural components of the sarcomere, namely, S and W for the cross-bridges thick filaments, while states B and CaTRPN for the myosin thin filament components. The remaining two states represent a conceptual component of the model related to the amount of distortion happening in the cross-bridges. There is also a seventh implicit state, U, that represents the fraction of unbound cross-bridges after subtracting all the cross-bridges that are in the strongly bound (S) and weakly-bound (W) from the available unblocked binding sites on actin (1- B) states.

**Fig. 1:**
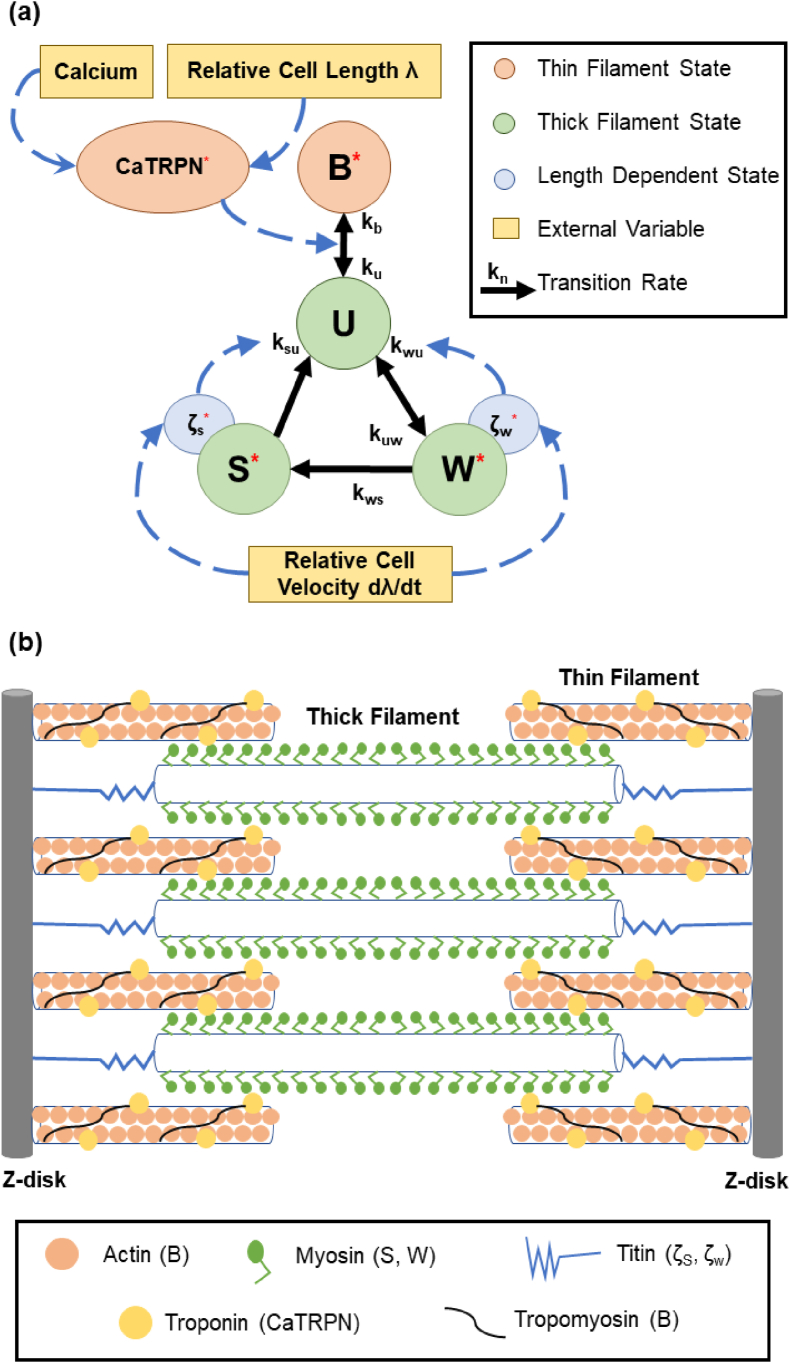
(A) Fractional Land Model Representation with transition rates and influences between states. The red asterisks indicate the states defined by Caputo's Fractional Derivatives. (b) Schematics of the cardiac sarcomere showing thick and thin filament proteins in relation to the CLS states.

Three of these seven states (S, W, and U) represent the cross-bridge model, which advances to the two-state Recruitment-Decay models [15,56]. Furthermore, states B and CaTRPN capture thin filament interactions, while the remaining two states capture length-dependent interactions. Land's model takes two main inputs: calcium concentration and relative sarcomere length. Fig. 1a illustrates these different states, which generate the active tension at the cell level, and how they interact with each other. The asterisks in the figure indicate all states explored to benefit from having their time derivatives re-expressed as Caputo's fractional derivatives; the Results section then identifies when using an FDE expression is significant compared to the original ODE expression for each state. The coefficients (*k*'s) on the black arrows in the Figure designate the transition rates between states. The transition rates are herein the same as in the original Land model. They are derived based on the velocity of tension development in healthy cardiac samples [22]. Fig. 1b shows a structural representation of the sarcomere, emphasizing the most prominent protein structures that contribute to a successful power stroke and their correlation with the CLS states.

Next, we revise Land's Model using the fractional derivative for all its six equations. The resulting FDE system (CLS) becomes as follows,

(3) DaCtαsS=kwsW−ksuS−γsuS

(4) DaCtαwW=kwsU−kwuW−kwsW−γwuW

(5) DaCtαZsζs=Asdλdt−csζs

(6) DaCtαZwζw=Awdλdt−cwζw

(7) DaCtαBB=kb*CaTRPN−nTm2*U−ku*CaTRPNnTm2*B

(8) DaCtαCaCaTRPN=kTRPN(([Ca2+]i[Ca2+]T50)nTRPN*(1−CaTRPN)−CaTRPN)

The subscripts in Equations (3), (4), (5), (6), (7), (8)) refer to their respective system's state. Moreover, k represents the transition rate from one state to the other, for example, k_ws_ refers to the transition rate between the pre-power stroke, W, and the post-power stroke, S, state. In equations (3), (4)), γ represents the cross-bridges distortion-dependent unbinding rates. In equations (5), (6), A represents the response-to-distortion amplitude, λ represents the relative length of the sarcomere during the quick stretch activation period and c represents the distortion decay rate for both distortions. In equation (8), [Ca^2+^]_i_ represents the initial concentration of calcium ions in the sarcomere and [Ca^2+^]_T50_ represents the calcium ion half activation point based on a human calcium transient provided by Ref. [57]. The nTm and nTRPN exponents represent the cooperativity coefficients that represent the thin filament contribution to the power stroke.

### 2.3 Numerical implementation

A coupled FDE numerical solver is needed to solve equations (3), (4), (5), (6), (7), (8) simultaneously as a system. The Predictor-Corrector (PC) method is a successful and inexpensive approach to solving similar FDE systems. An FDE predictor-corrector algorithm converts the fractional order differential equation (based on Caputo's definition) into a corresponding Volterra integral equation, which can be solved by the predictor-corrector method. Generally, the predictor part of the algorithm starts by guessing a solution that solves the problem, followed by the corrector, which refines the solution through iterations that minimize integration error [58]. Specifically, we will follow the definitions by Garrappa [59].

Accordingly, the predicted state variable *y*^*P*^ at time increment *n* is defined by equation (9) as:

(9) $$y_{n}^{P}=y_{0}+h^{\alpha }\sum_{j=0}^{n-1}\frac{{\left(n-j\right)}^{\alpha }-{\left(n-j-1\right)}^{\alpha }}{\Gamma \left(\alpha +1\right)}f_{j}$$

Where *y*_0_ is the initial value of the state at the start of the model, *α* is the order of the fractional derivative, *h* is the step size, and Γ is Euler's gamma function, and f_j_ designates a state variable's first derivative evaluated at time increment t_j_. The corrected $y_{n}$ is then defined by equation (10) as:

(10) $$y_{n}=y_{0}+h^{\alpha \;}a_{n,0}f_{0}+h^{\alpha \;}\sum_{j=1}^{n-1}\beta_{n-j}{\;f}_{j}+h^{\alpha \;}\beta_{0\;}f\left(t_{n},y_{n}^{P}\right)\text{,}$$

where $\beta_{n}$ and $a_{n,0}$ are respectively defined by equations (11), (12) as,

(11) $$\beta_{n}=\left\{ \begin{array}{c} \frac{1}{\Gamma \left(\alpha +2\right)}\;n=0,\\ \frac{{\left(n-1\right)}^{\alpha +1}-2\;n^{\alpha +1}+{\left(n+1\right)}^{\alpha +1}}{\Gamma \left(\alpha +2\right)}\;n=1,2,\ldots \end{array}\right.$$

(12) $$a_{n,0}=\frac{{\left(n-1\right)}^{\alpha +1}-n^{\alpha }\;\left(n-\;\alpha \;-1\right)}{\Gamma \left(\alpha +2\right)}$$

### 2.4 CLS calibration for cardiomyocytes

**Fig. 2:**
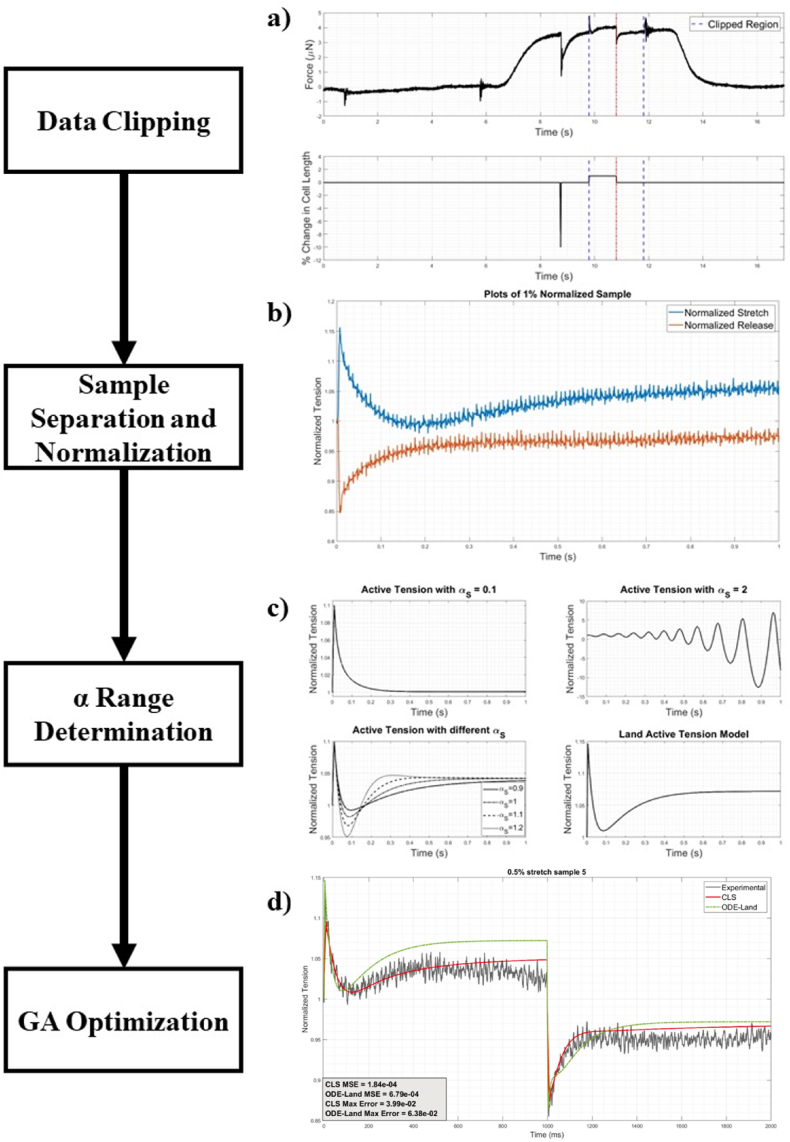
Summary of the proposed CLS calibration steps. (a) Designates data clipping between the vertical dotted lines, (b) represents the partitioning of the curve into stretch and release phases, (c) represents the determination of fractional order range depending on mathematical stability, and (d) represents the use of GA optimization of the fractional orders to capture measured stretch and release behaviors.

Determining the fractional order (α) of each state equation in the CLS is important to understand the role of non-locality in the processes that underlie evolving state variables and, ultimately, in the active tension modeling of cardiomyocytes. The dataset used to calibrate the CLS is based on Land's quick stretch experiments, which record the tensile force response in a cardiac cell due to step-like mechanical perturbations at a specific calcium level, cf [22,60]. The dataset includes 21 skinned healthy human left ventricular myocardium samples obtained from Ref. [61]. This set is divided into 5 samples at 0.5% stretch, 11 samples at 1% stretch, and 5 samples at 2% stretch. The steps taken to calibrate our proposed CLS based on this dataset are summarized in Fig. 2.

The model is thus calibrated using skinned cells, which we remark have some different viscoelastic properties from intact cells. Specifically, there is evidence that the skinning procedure affects the outer layer of the cell membrane, which results in some changes to calcium cooperativity, sensitivity and maximal active tension in the cell [22,62]. There is no evidence that the skinning process affects the cytoskeletal contribution to viscoelasticity. Skinning alterations to viscoelasticity can be corrected for by changing the [Ca^2+^]_T50_, nTm, k_uw,_ and k_ws_ variables of the model to accurately account for the difference between skinned and intact cells. Indeed, both Land's and ToR's models in the literature have parameterized these variables to successfully model intact cell behavior [63]. The CLS model proposed herein, therefore, could in the same manner be extended to intact cell behavior.

#### 2.4.1 Data clipping

The original dataset includes continuous tensile waveforms from pre-activation, activation, and relaxation periods associated with the quick stretch and release protocol. The relevant data segment to our model is the 2-s interval where the quick stretch and release step-like perturbation was performed during the activation period. Fig. 2a (reproduced from Ref. [22] based on a 1% stretch complete protocol) shows the original waveform for the input stretch signal and output force measurements. We clip the 2-s interval between the vertical blue lines shown. The red dotted line indicates the stretch segment's end and the release segment's beginning.

#### 2.4.2 Sample separation and normalization

The clipped 2-s interval is then separated into stretch and release segments based on the initial stretch signal and normalized to provide a standard frame of reference to compare the original and fractional Land models to the experimental data. Fig. 2b illustrates a case for the 1% protocol separated into stretch and release segments. Our proposed separation of the 2-s interval into stretch and release segments has been adopted in previous models to better represent their dissimilar mechanics [15]. Specifically, such separation may be justified by differences in the stretch and release mechanisms as seen from the ATP and cross-bridge cycling point-of-view. The sliding distance by the thin filament induced by cross-bridges during the ATP hydrolysis cycle differs during a power stroke depending on the existence of a load [64]. In Land's model, the stretch part signifies the existence of a tensile load, and the release part is the absence of such load.

#### 2.4.3 α-Range determination

To determine the permissible range for each α, which maintains system stability during CLS optimization, we first change its values between wide limits, i.e., 0.1 to 2, at increments of 0.1, while holding all the other α′s fixed at 1. The choice of limiting the α′s between 0.1 and 2 is based on a recent study that suggests that the viscoelastic behavior in striated muscle cells does not only depend on current cell length and stretch velocity but also the acceleration of the stretch [65]; i.e., system states can depend on zeroth, first and/or second-order time derivatives. Specifically, a multi-order systems FDE solver [66], which we adapted to Land's MATLAB code [61], is here used to solve the CLS. System stability is then enforced by following the tensile waveform generated for different α values and ensuring that the mechanical response is not underdamped or overdamped. Fig. 2c illustrates various active tension waveforms for the case of α_s_. With these permissible subranges of α obtained, CLS optimization ensues.

#### 2.4.4 Genetic Algorithm (GA) optimization

For the stretch segment, the CLS is started with the initial steady state (SS) conditions based on the initial fraction of formed cross-bridges, initial calcium concentration, and the stretch protocol obtained from the Land codebase [61]. The multi-order systems solver then advances the solution of the CLS states at 0.05-ms intervals per the following α_i_ vector (equation (13)),

(13) $$\alpha_{i},\forall i\in \left\{S,W,\text{CaTRPN},B,\zeta_{s},\zeta_{w}\right\}$$

until the system states are determined for the first second of the quick stretch protocols. Accordingly, the active tension (T_active_) is determined by (equation (14)),

(14) $$T_{active}=L_{fac}\left(\left(\zeta_{s}+1\right)S+\zeta_{w\;}W\right)$$

Where *L*_*fac*_ is the length-dependent factor based on the sample's initial sarcomere length and the reference maximal tension. The mean-square-error (MSE) between the model-calculated active tension and that of the experimental sample is the qualifier we used to measure whether the model is the best fit based on the current α_i_ vector.

The MATLAB Genetic Algorithm (ga) code, part of the Global Optimization Toolbox [67], is then used to optimize fractional state orders based on MSE measurements. Selected algorithm parameters are summarized in Table 1. Algorithm parameters missing from Table 1, such as crossover and mutation parameters, are unchanged from the algorithm's default values in Ref. [68].

**Table 1:** GA parameters.

Parameter	Value
α Lower Bound	Stretch [0.9,0.1,0.8,0.1,0.1,0.1]
	Release [0.1,0.1,0.8,0.1,0.6,0.5]
α Upper Bound	Stretch [1.3,1.6,2.0,2.0,1.4,1.3]
	Release [1.2,1.5,1.8,1.9,1.4,1.3]
MSE Function Tolerance	1e-5
Population Size	50
Max Generation number	700
Initial Population, α_i_	[1,1,1,1,1,1]

**Fig. 3:**
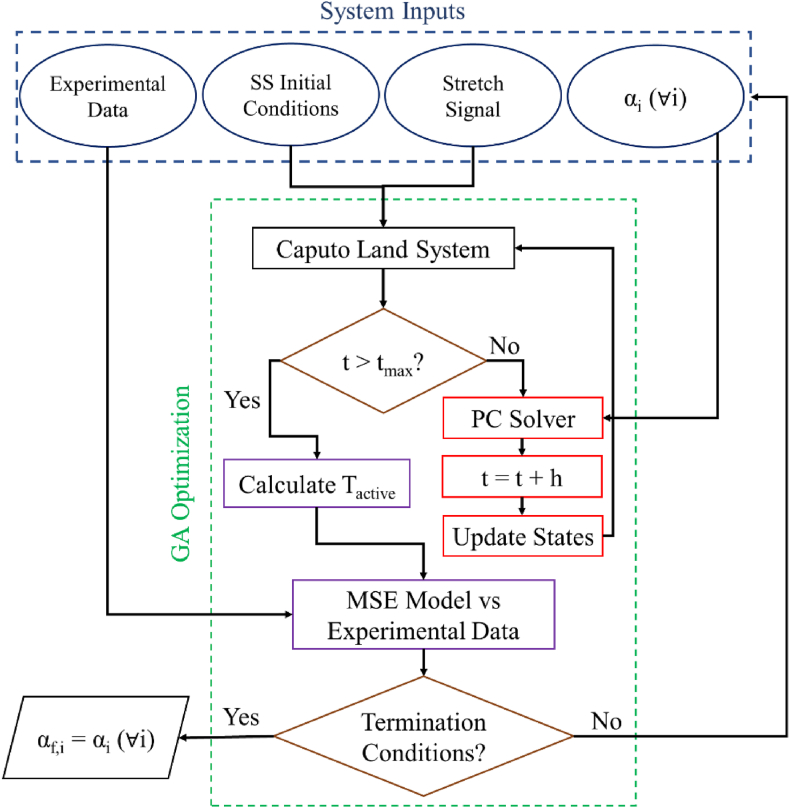
Summary of the GA optimization workflow for the stretch segment. t_max_ represents the upper time limit (1 s) for each segment, and h represents the solver step size (0.05 ms).

In Table 1, MSE Function tolerance and Max Generation number refer to the criteria for ga-termination; the optimization process continues until either the MSE is less than this tolerance or the maximum number of generations is reached. At the end of GA optimization, the α_f,i_ vector represents the final order of the system's states and is set to the α_i_ vector when the system has reached its termination condition. A flow chart of this GA optimization (for quick stretch and release) is shown in Fig. 3.

The steady-state results at the end of the stretch segment are then used as the initial condition for the release segment (hence our need for the Caputo derivative). This computational model is thus reapplied to obtain the active tension curve for the release segment. Stretch and release model segments are then reconsolidated into a single graph to be compared to the experimental results and Land's original model, as shown in Fig. 2d.

## 3 Results and discussion

This section first examines the CLS's ability to model cardiomyocyte active tension (variability) for twenty-one experimental samples. It next analyzes the mechanisms of viscoelasticity at the cell level, which can explain the modeled active tension.

### 3.1 Capturing active tension and its variability

**Fig. 4:**
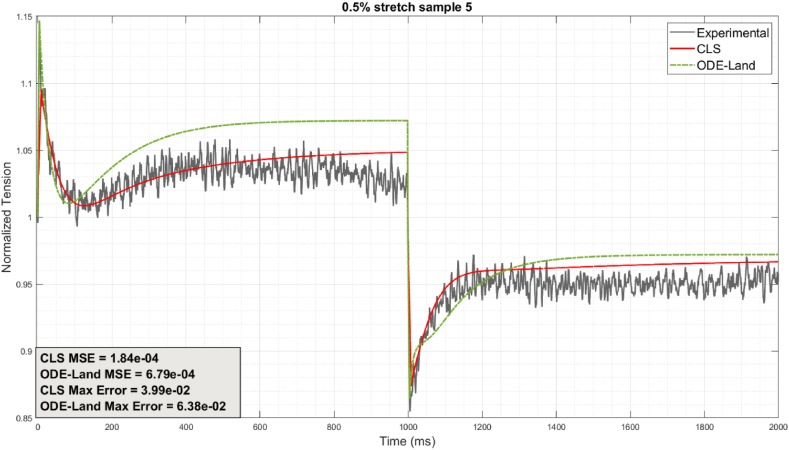
Comparison between CLS and ODE-Land individual sample fit.

The fractional orders of our proposed CLS are fitted to the experimental samples individually using the algorithm summarized in Fig. 3, then compared to Land's reference model and experiments. Fig. 4 shows the results we obtained for a 0.5% stretch sample. By visual inspection, our CLS's accounting for viscoelasticity (via fractional orders) permits a significantly improved correspondence with the experiment than the reference Land model, especially during the first 1400 ms of the cycle, where notable force changes are exhibited. Moreover, the CLS's maximum and mean-square errors (MSE) confirm that the CLS consistently outperforms Land's model for the duration of the 2-s stretch and release protocols.

**Fig. 5:**
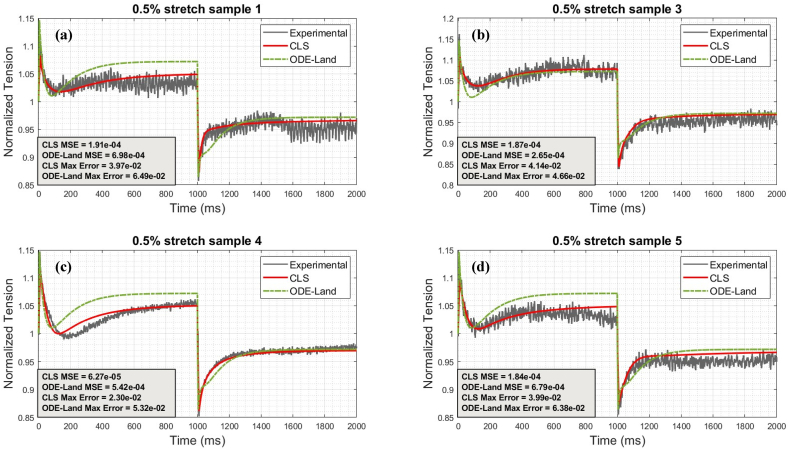
Comparison between CLS and ODE-Land models for representative 0.5% protocol samples: (a) represents sample 1, (b) represents sample 3, (c) represents sample 4, and (d) represents sample 5.

**Fig. 6:**
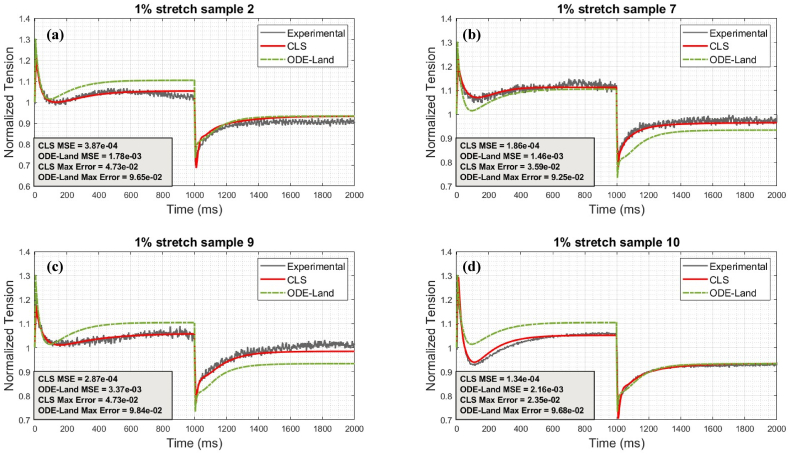
Comparison between CLS and ODE-Land models for representative 1% protocol samples: (a) represents sample 2, (b) represents sample 7, (c) represents sample 9, and (d) represents sample 10.

**Fig. 7:**
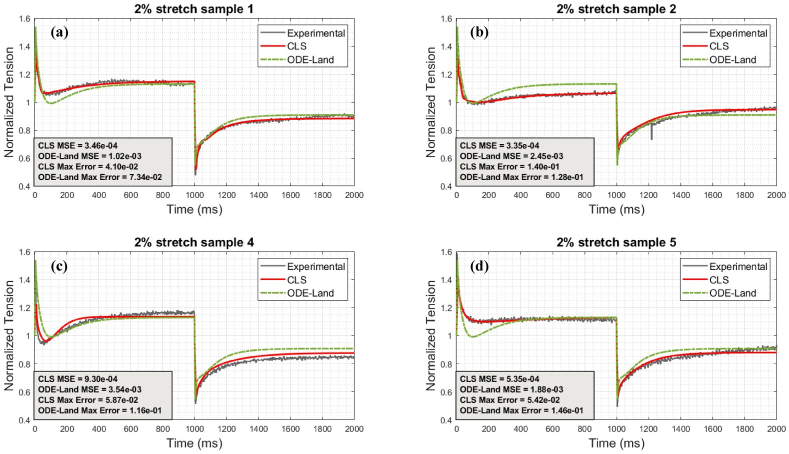
Comparison between CLS and ODE-Land models for representative 2% protocol samples: (a) represents sample 1, (b) represents sample 2, (c) represents sample 4, and (d) represents sample 5.

Fig. 5, Fig. 6, Fig. 7 illustrate selected samples from the 0.5%, 1%, and 2% protocols. It is again confirmed that the CLS generally outperforms the reference Land model in matching individual cases and thus promises greater subject-specificity. Tables A1-A3 in Appendix A summarize the error results for all protocol samples. The average CLS MSE and maximum errors for all samples are consistently less than their counterparts for the reference model.

**Fig. 8:**
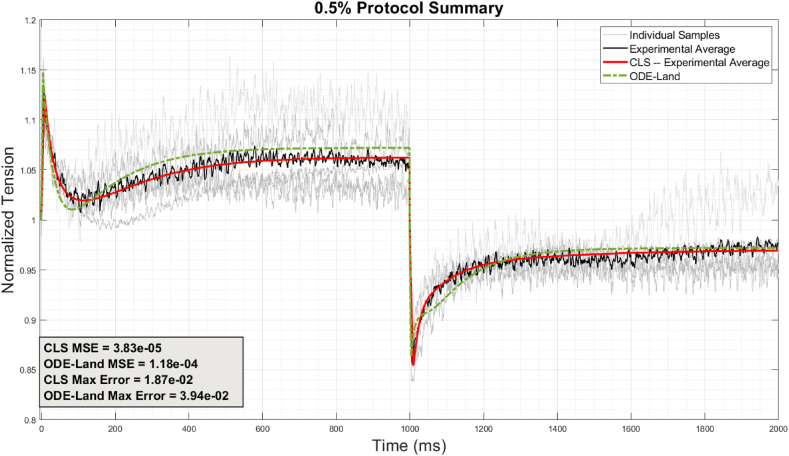
Experimental samples and average represented by CLS and ODE-Land models for 0.5% stretch samples.

Fig. 8 shows the CLS's representation of the average for the 0.5% stretch protocols. The grey traces represent the individual experimental samples, while the black trace designates their arithmetical average. The red line indicates the CLS's fit, while the green dashed line is the reference Land model. Again, by visual inspection and numerically, the CLS outperforms the reference model, which confirms the importance of capturing viscoelasticity for the average behavior, not just explaining the variations across a population.

**Fig. 9:**
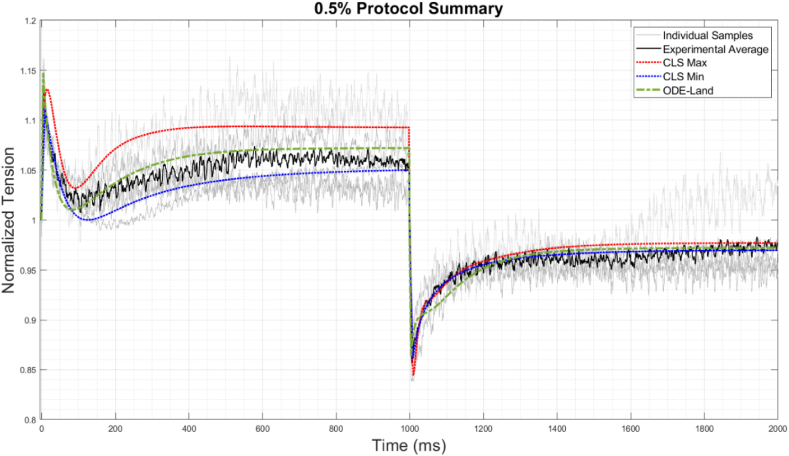
Comparison among ODE-Land, maximum and minimum CLS superimposed on the individual 0.5% stretch samples.

Nevertheless, viscoelasticity, as modeled by our CLS, does not explain all the variability in active tension across a population; see Fig. 9, which illustrates the results for the 0.5% protocol and the range that the CLS could cover based on the fractional orders listed in Tables A4 and A5 in Appendix A. The blue and red traces indicate the minimum and maximum of the CLS range, while the green dashed line designates the reference model fitted to the same experiments. As seen in the Figure, the CLS captures a good range of variability in the stretch phase but not very much in the release phase and becomes especially insensitive after the 1400 ms mark. This is consistent with the notable changes to the tension curve that occur during the first 1400 ms (which plateaus afterward), suggesting a greater rate-dependency of the mechanisms involved [64] and greater relevance to viscoelasticity in that time range. We note that sample two has been shaded with a lighter grey in both Fig. 8, Fig. 9 to indicate its obvious deviation in trend and magnitude from all the other samples. Our model cannot fully explain its deviation, specifically after 1400 ms, which could be attributed to experimental peculiarity. Nevertheless, as noted earlier, the CLS captures its behavior significantly better than the reference model, so viscoelasticity offers a partial explanation.

**Fig. 10:**
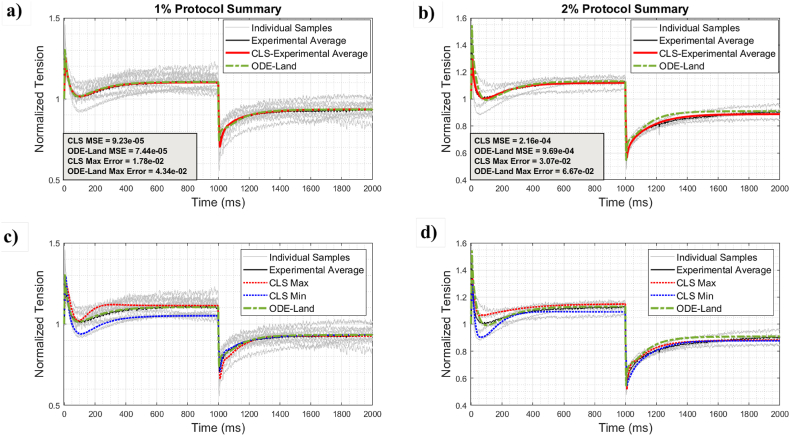
Comparison among ODE-Land, maximum and minimum CLS superimposed on the individual 1% (left) and 2% (right) stretch sample traces.

Fig. 10a and b portray the results for 1% and 2% stretch protocols, respectively, while Fig. 10c and d contrast the CLS maximum and minimum to the reference model. Again, the findings confirm that the CLS provides a more accurate representation than the reference. Specifically, for the 1% protocol, the CLS average curve is on par with the reference. However, the maximum and minimum of its range allow for a better match to the individual samples. For the 2% protocol, the CLS performs better than the reference, even in the release phase.

### 3.2 Identification of cell-level states that contribute to viscoelasticity

We summarize in Table 2 cross-bridge interactions (thick filament) and length-dependent interactions that we identified in the CLS to directly influence active tension generation, per Equation (14). Thin filament interactions implicitly influence active tension generation by altering the time evolution of the thick filament interactions. There have been many studies in the literature that debate whether cytoskeleton constituents are inherently viscoelastic or whether some contribute structurally to the overall viscoelasticity of the cell [25]. Some studies have concluded that the cell's cytosol exhibits viscoelastic behavior [69,70], while others have concluded that the contribution of the cytoskeleton to viscoelasticity results from constituent interactions, such as myosin cross-bridges [71], and actin-binding proteins [72]. Indeed, of the six states modeled by the CLS, those known in the literature as potential viscoelastic contributors are indicated in Table 2.

**Table 2:** Summary of state influence on other states and on active tension.

Changes in State	Main Interaction	Directly Influences	Influenced by	Viscoelastic Contributor [Ref.]
**S**	Thick Filament	Active Tension	S, W, ζ_s_	Yes [71]
**W**	Thick Filament	Active Tension	B, S, W, ζ_w_	Yes [71]
**CaTRPN**	Thin Filament	B	CaTRPN, λ	–
**B**	Thin Filament	W	CaTRPN, S, B, W	Yes [73]
**ζ**_**s**_	Length Dependent	S, Active Tension	λ, ζ_s_	–
**ζ**_**w**_	Length Dependent	W, Active Tension	λ, ζ_w_	–

Table 3, on the other hand, summarizes the states' sensitivity based on the CLS. High fractionality means |α−1|>0.2, i.e., the fractional order for the state is at least 20% away from the first derivative (reference model), which we deem indicates significant viscoelasticity. The CLS thus confirms the W and B states are viscoelastic contributors, as noted in the literature. It further reveals the CaTRPN state as a potential contributor and does not find the S state to be a significant contributor, which warrants further experimental research. Interestingly, while state S does not portray high viscoelasticity based on the provided criteria, small variations in its α-value do result in a significant difference in the tension development, so a fractional representation of state S is still relevant to the model, which speaks the system's strong nonlinearity in S. On the other hand, the effect of changing states ζ_s_ and ζ_w_ does not provide drastic changes in the tension development, suggesting that length-dependent viscoelastic contributions are not prominent, and integer order derivatives will suffice for them.

**Table 3:** State α sensitivities based on trends seen from experimental calibration.

State	α Fractionality
**Post Power Stroke (S)**	–
**Pre-Power Stroke (W)**	High
**CaTRPN**	High
**B**	High
${\zeta \;}_{s}$	–
${\zeta \;}_{w}$	–

Interpreting these findings from the perspective of CLS's potential to support the future of in-vitro diagnostics and drug screening, we see that it excludes two of the six state variables (i.e., length-dependent interactions) from further analysis of the observed inter-subject variability in mechanical behavior. It thus serves to focus in-vitro studies of the relevant disease/drug kinetics. Moreover, for this dataset, the CLS prioritizes a further analysis of the S state over the W, CaTRPN, and B states, since it strongly alters thick-filament interactions and apparent cellular viscoelasticity with small a variation in *α* (from integer order). Molecular investigations into S-state kinetics could reveal how a little viscoelasticity yields visible behavioral change in the cell.

The proposed CLS has thus improved the capabilities of Land's system in capturing inter-subject variabilities in cardiac viscoelasticity. This is to be expected, given the literature on factional calculus, e.g., Ref. [33]. The advantage of resorting to FDEs in general lies in their ability to greatly reduce a cellular system's modeling complexity and to yield quick results that allow further focusing and guidance of molecular-level investigations into the identified interactions (those most sensitive to α).

## 4 Conclusion and future work

This research showed that our proposed Caputo-Land System (CLS) can effectively describe cardiac viscoelastic behavior and identify its underlying cell-level mechanisms. The CLS was used to effectively characterize the active tension curves for experimental quick stretch and release experiments 0.5%, 1%, 2% stretch protocols. It identified three state variables as particularly viscoelastic, of which CaTRPN is new to the literature. Moreover, it finds the dynamical system strongly nonlinear in state S, though the state is not extensively viscoelastic, suggesting a disproportionate influence on observed cell behavior, and drawing attention toward the need for its future investigation. The results of our CLS also outperformed the reference state-of-the-art model in terms of subject-average behaviors, as well as an ability to better match individual behaviors, promising subject-specificity. The CLS system in this research has only been applied to skinned healthy cardiac cells, which limits its current coverage of possible viscous cellular mechanisms; however, the model has the potential to be applied to intact diseased samples taken from patients, by recalibrating a few definite parameters to available data. As such, the CLS system can elucidate in-silico the influence of viscoelastic mechanisms on observed cell mechanics (e.g., obtained in-vitro using biosensors) to help diagnose various diseases [74], as well as to screen drugs and evaluate them, e.g., for cardiotoxicity [75].

The CLS can be trained on new quick stretch and release experiments with different degrees of disease to see the effects of the changing viscoelastic behavior on α′s and allow for a more comprehensive model for healthy and diseased cells. To the authors' knowledge, there is no open-source data for diseased human cardiac cells based on quick stretch and release experiments, which can be a focus point in future studies. Future studies can also benefit from applying different types of fractional derivative definitions (other than Caputo) to investigate model accuracy and time efficiency, especially at the plateau region of the release phase where the present CLS system produced no significant improvements.

## Data availability

Data will be made available upon reasonable request.

## CRediT authorship contribution statement

**Afnan Elhamshari:** Writing – original draft, Visualization, Validation, Methodology, Formal analysis, Data curation. **Khalil Elkhodary:** Writing – review & editing, Writing – original draft, Supervision, Methodology, Funding acquisition, Formal analysis, Conceptualization.

## Declaration of competing interest

The authors declare that they have no known competing financial interests or personal relationships that could have appeared to influence the work reported in this paper.

## Figures and Tables

**Table A1:** Comparison between the CLS and ODE-Land errors from the 0.5% samples

Sample	CLS MSE	ODE-Land MSE	CLS Max Error	ODE-Land Max Error
1	1.91E-04	6.98E-04	3.97E-02	6.49E-02
2	7.39E-04	1.28E-03	9.09E-02	9.62E-02
3	1.87E-04	2.65E-04	4.14E-02	4.66E-02
4	6.27E-05	5.42E-04	2.30E-02	5.32E-02
5	1.84E-04	6.79E-04	3.99E-02	6.38E-02
Max Error	7.39E-04	1.28E-03	9.09E-02	9.62E-02
Average Error	2.73E-04	6.93E-04	4.70E-02	6.49E-02

**Table A2:** Comparison between the CLS and ODE-Land errors from the 1% samples

Sample	CLS MSE	ODE-Land MSE	CLS Max Error	ODE-Land Max Error
1	2.05E-04	6.45E-04	3.97E-02	7.77E-02
2	3.87E-04	1.78E-03	4.73E-02	9.56E-02
3	7.31E-04	7.57E-04	5.67E-02	6.27E-02
4	5.91E-04	1.52E-03	6.72E-02	7.65E-02
5	6.55E-04	7.35E-04	9.36E-02	9.21E-02
6	2.28E-03	3.07E-03	1.16E-01	1.55E-01
7	1.86E-04	1.46E-03	3.59E-02	9.25E-02
8	4.19E-03	6.12E-03	1.20E-01	1.50E-01
9	2.87E-04	3.37E-03	4.73E-02	9.84E-02
10	1.34E-04	2.16E-03	2.35E-02	9.68E-02
11	4.39E-04	2.20E-03	3.53E-02	1.06E-01
Max Error	4.19E-03	6.12E-03	1.20E-01	1.55E-01
Average Error	1.05E-03	2.33E-03	6.75E-02	1.02E-01

**Table A3:** Comparison between the CLS and ODE-Land errors from the 2% samples

Sample	CLS MSE	ODE-Land MSE	CLS Max Error	ODE-Land Max Error
1	3.46E-04	1.02E-03	4.10E-02	7.34E-02
2	3.35E-04	2.45E-03	1.40E-01	1.28E-01
3	1.02E-03	3.75E-03	6.64E-02	1.79E-01
4	9.30E-04	3.54E-03	5.87E-02	1.16E-01
5	5.53E-04	1.88E-03	5.42E-02	1.46E-01
Max Error	1.02E-03	3.75E-03	1.40E-01	1.79E-01
Average Error	6.37E-04	2.53E-03	7.21E-02	1.28E-01

**Fig. A1:**
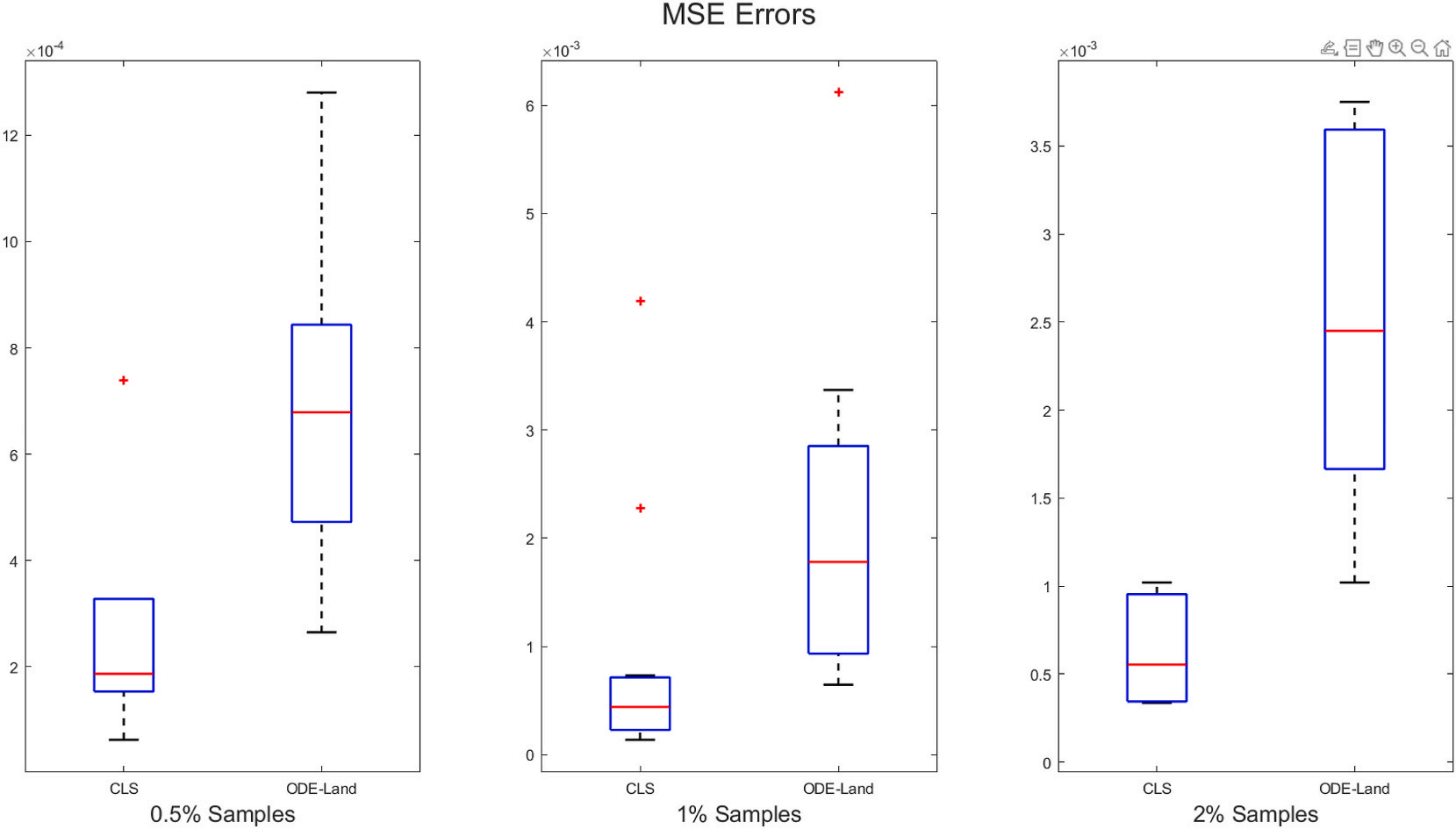
Comparison between MSE errors for both models.

**Fig. A2:**
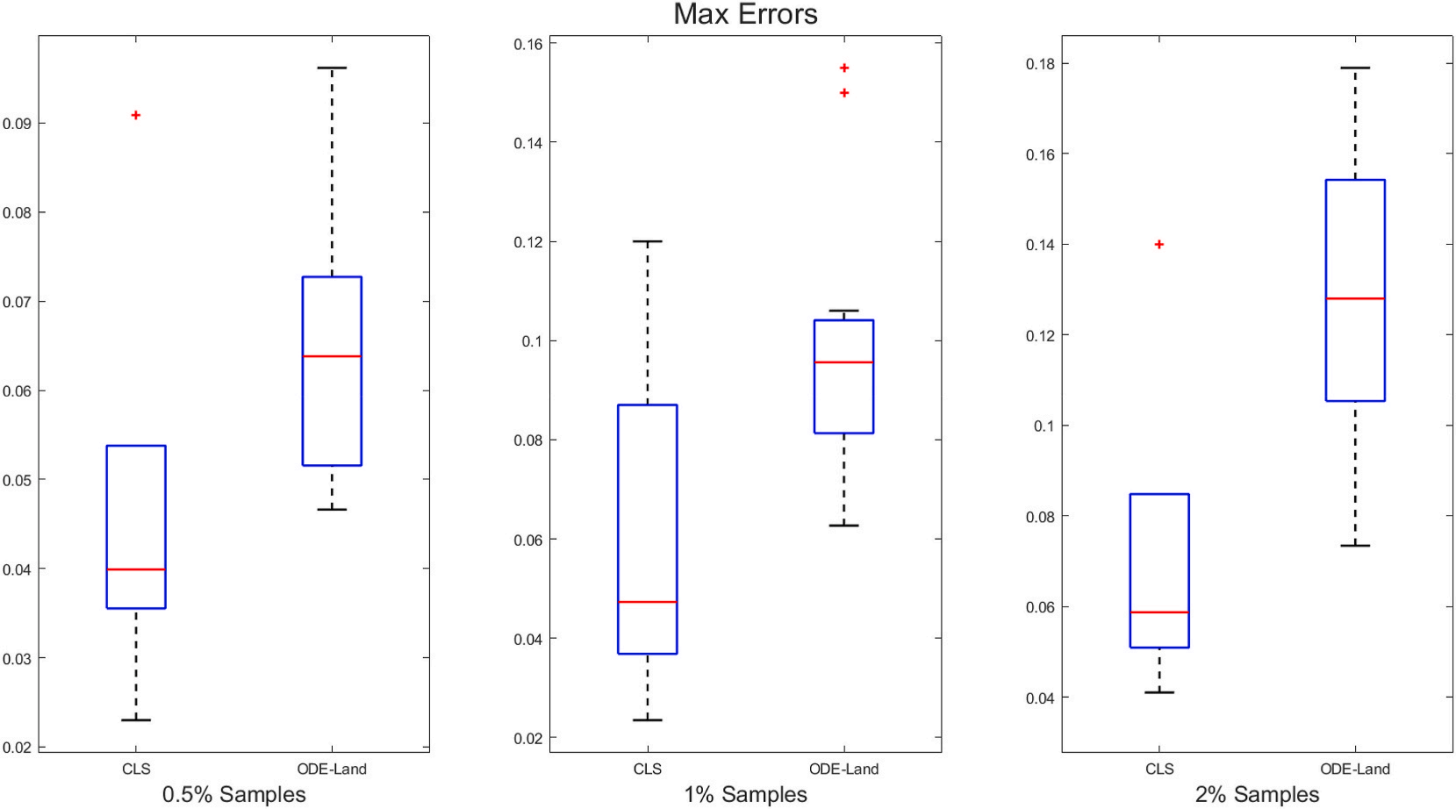
Comparison between Maximum errors for both models.

**Table A4:** Values of each state order (α) for the 0.5% stretch segments

	Sample	α S	α W	α TRPN	α B	α $\zeta_{s}$	α $\zeta_{w}$
CLS RESULTS	**1**	0.9	0.9	1.9	0.2	1.0	0.5
	**2**	1.1	0.1	1.2	2.0	1.3	0.1
	**3**	1.0	0.8	1.6	1.8	1.0	0.7
	**4**	0.9	0.8	1.0	1.1	1.2	0.8
	**5**	0.9	1.2	1.9	1.0	1.2	0.5
	**experimental average**	1.0	0.6	1.8	0.5	1.0	1.0
STATISTICAL ANALYSIS	**α average**	0.97	0.76	1.54	1.22	1.12	0.54
	**α STD**	0.07	0.36	0.36	0.65	0.13	0.23

**Table A5:** Values of each state order (α) for the 0.5% release segments

	Sample	α S	α W	α TRPN	α B	α $\zeta_{s}$	α $\zeta_{w}$
CLS RESULTS	**1**	0.7	0.6	1.4	0.2	0.8	1.1
	**2**	1.0	0.9	1.4	0.8	1.2	1.3
	**3**	0.9	1.1	1.0	0.2	1.1	1.2
	**4**	1.1	0.5	1.4	1.5	1.1	1.0
	**5**	0.8	0.6	1.4	1.0	1.1	0.7
	**experimental average**	0.9	0.8	1.5	0.8	1.1	1.2
STATISTICAL ANALYSIS	**α average**	0.88	0.76	1.31	0.71	1.07	1.07
	**α STD**	0.14	0.21	0.17	0.51	0.13	0.19

**Table A6:** Values of each state order (α) for the 1% stretch segments

	Sample	α S	α W	α TRPN	α B	α $\zeta_{s}$	α $\zeta_{w}$
**CLS** RESULTS	**1**	1.2	0.3	1.7	1.3	0.9	1.0
	**2**	1.0	0.5	1.0	0.2	1.0	1.0
	**3**	1.1	0.7	1.4	1.8	1.0	1.2
	**4**	1.3	0.2	1.2	1.8	1.0	0.8
	**5**	1.1	1.4	1.3	2.0	1.0	0.9
	**6**	1.2	0.1	1.0	2.0	1.2	0.3
	**7**	1.1	0.2	1.0	1.9	1.0	0.7
	**8**	1.2	0.1	1.9	2.0	1.0	1.0
	**9**	0.9	1.0	1.8	0.3	1.0	0.6
	**10**	1.0	0.8	1.3	1.0	1.2	1.3
	**11**	1.0	0.8	1.0	1.0	1.1	1.3
	**experimental average**	1.0	1.5	1.8	1.0	1.1	0.9
STATISTICAL ANALYSIS	**α average**	1.11	0.57	1.33	1.39	1.04	0.91
	**α STD**	0.11	0.41	0.32	0.64	0.11	0.29

**Table A7:** Values of each state order (α) for the 1% release segments

	Sample	α S	α W	α TRPN	α B	α $\zeta_{s}$	α $\zeta_{w}$
**CLS** RESULTS	**1**	0.8	1.2	1.2	0.2	1.0	1.3
	**2**	1.0	0.7	1.5	0.8	1.1	1.3
	**3**	1.0	0.8	1.7	1.0	1.2	0.7
	**4**	1.1	0.6	1.5	1.3	1.0	1.2
	**5**	1.1	1.0	1.0	0.2	1.2	1.3
	**6**	1.2	0.7	1.2	0.4	1.2	1.3
	**7**	0.8	1.2	1.1	1.6	1.0	1.2
	**8**	1.2	0.9	1.6	1.1	1.3	0.8
	**9**	1.0	0.9	1.8	0.7	1.0	1.2
	**10**	0.9	0.8	1.5	0.9	1.1	1.2
	**11**	1.0	0.7	1.4	0.6	1.0	1.1
	**experimental average**	1.1	0.7	1.2	0.7	1.1	1.2
STATISTICAL ANALYSIS	**α average**	1.00	0.87	1.41	0.78	1.11	1.14
	**α STD**	0.12	0.19	0.25	0.43	0.11	0.19

**Table A8:** Values of each state order (α) for the 2% stretch segments

	Sample	α S	α W	α TRPN	α B	α $\zeta_{s}$	α $\zeta_{w}$
**CLS** RESULTS	**1**	1.0	1.5	0.9	1.8	0.9	1.2
	**2**	0.9	1.5	0.9	1.6	1.0	1.3
	**3**	1.1	0.9	0.9	1.1	0.1	1.3
	**4**	1.2	1.6	1.1	1.7	0.3	0.7
	**5**	0.9	0.2	1.9	0.3	0.8	0.9
	**experimental average**	1.0	1.3	0.9	1.7	0.7	0.8
STATISTICAL ANALYSIS	**α average**	1.02	1.14	1.11	1.30	0.61	1.09
	**α STD**	0.10	0.53	0.39	0.56	0.34	0.23

**Table A9:** Values of each state order (α) for the 2% release segments

	Sample	α S	α W	α TRPN	α B	α $\zeta_{s}$	α $\zeta_{w}$
CLS RESULTS	**1**	0.9	0.9	1.7	0.2	1.0	1.3
	**2**	1.0	0.7	1.3	0.9	1.0	0.8
	**3**	1.1	0.8	1.3	1.0	1.2	0.8
	**4**	1.0	0.7	1.4	0.8	1.1	0.8
	**5**	1.0	0.8	1.7	0.8	1.1	0.9
	**experimental average**	1.0	0.8	1.3	0.8	1.1	0.9
STATISTICAL ANALYSIS	**α average**	1.02	0.81	1.48	0.74	1.07	0.92
	**α STD**	0.05	0.07	0.19	0.27	0.05	0.19

## Appendix A

Tables A1-A3 show the results of the MSE and max errors for both the CLS and the ODE-Land models for 0.5, 1, and 2% stretch protocols. In all protocols, the average CLS MSE and Max error is lower than those of the original Land model. Figures A1 and A2 show the statistical comparison between MSE and maximum errors, respectively, for both models. In all subplots the median of the boxplot is always lower for the CLS compared to the ODE-Land model, confirming a better fit to FDEs compared to ODEs.

Tables A4-A9 show the order of each FDE state based on the sample and the stretch protocol (0.5, 1, 2% respectively). As mentioned before, the α′s are fitted differently to stretch and release segments and the results are reported accordingly. α average represents the average of all the sample α for that state according to N, the number of samples in the protocol, and α STD represents the spread of α average.

(16) $$\alpha_{i,average}=\frac{\sum_{j=1}^{N}\alpha_{ij}}{N},\forall i\in \left\{S,W,\text{CaTRPN},B,\zeta_{s},\zeta_{w}\right\}$$

(17) $$\alpha_{i,STD}=\sqrt{\frac{\sum_{j=1}^{N}\left(\alpha_{ij}-\alpha_{i,average}\right)}{N}},\forall i\in \left\{S,W,\text{CaTRPN},B,\zeta_{s},\zeta_{w}\right\}$$
